# ER translocon inhibitor ipomoeassin F inhibits triple-negative breast cancer growth via blocking ER molecular chaperones

**DOI:** 10.7150/ijbs.82012

**Published:** 2023-07-31

**Authors:** Shishi Tao, Eun Ju Yang, Guanghui Zong, Pui Kei Mou, Guowen Ren, Yue Pu, Liang Chen, Ho Jeong Kwon, Jianhong Zhou, Zhijian Hu, Arman Khosravi, Qingyang Zhang, Yuchun Du, Wei Q. Shi, Joong Sup Shim

**Affiliations:** 1 Cancer Centre, Faculty of Health Sciences, University of Macau, Taipa, Macau SAR, China.; 2 Department of Chemistry and Biochemistry, University of Maryland, College Park, Maryland 20742, USA.; 3 Shenzhen Laboratory of Tumor Cell Biology, Institute of Biomedicine and Biotechnology, Shenzhen Institute of Advanced Technology, Chinese Academy of Sciences, Shenzhen 518055, China.; 4 Chemical Genomics Leader Research Laboratory, Department of Biotechnology, College of Life Science and Biotechnology, Yonsei University, Seoul 03722, Korea.; 5 Department of Biological Sciences, University of Arkansas, Fayetteville, Arkansas 72701, USA.; 6 Feinstein Institute for Medical Research, Northwell Health, 350 Community Dr., Manhasset, New York, 11030, USA.; 7 Department of Chemistry, Ball State University, Muncie, Indiana 47306, USA.; 8 Department of Mathematical Sciences, University of Arkansas, Arkansas 72701, USA.; 9 MOE Frontiers Science Centre for Precision Oncology, University of Macau, Taipa, Macau SAR, China.

**Keywords:** Ipomoeassin F, ER translocon, TNBC, PDIA6, PDIA4, ER stress

## Abstract

Triple-negative breast cancer (TNBC) is an aggressive type of breast cancer where no effective therapy has been developed. Here, we report that the natural product ER translocon inhibitor ipomoeassin F is a selective inhibitor of TNBC cell growth. A proteomic analysis of TNBC cells revealed that ipomoeassin F significantly reduced the levels of ER molecular chaperones, including PDIA6 and PDIA4, and induced ER stress, unfolded protein response (UPR) and autophagy in TNBC cells. Mechanistically, ipomoeassin F, as an inhibitor of Sec61α-containing ER translocon, blocks ER translocation of PDIA6, inducing its proteasomal degradation. Silencing of PDIA6 or PDIA4 by RNA interferences or treatment with a small molecule inhibitor of the protein disulfide isomerases in TNBC cells successfully recapitulated the ipomoeassin F phenotypes, including the induction of ER stress, UPR and autophagy, suggesting that the reduction of PDIAs is the key mediator of the pharmacological effects of ipomoeassin F. Moreover, ipomoeassin F significantly suppressed TNBC growth in a mouse tumor xenograft model, with a marked reduction in PDIA6 and PDIA4 levels in the tumor samples. Our study demonstrates that Sec61α-containing ER translocon and PDIAs are potential drug targets for TNBC and suggests that ipomoeassin F could serve as a lead for developing ER translocon-targeted therapy for TNBC.

## Introduction

Breast cancer has surpassed lung cancer in the incidence rate and became the most common human malignancy in 2020^1^. Despite advanced technologies and new drugs in diagnosis and treatment, the incidence and mortality of global breast cancer patients continue to rise^2^, demonstrating the urgent need for novel therapeutic strategies. According to the molecular characteristics, such as hormone receptors (HR) and human epidermal growth factor receptor 2 (HER2) expression status, breast cancer can be divided into four molecular subtypes: luminal A (HR^+^/HER2^-^), luminal B (HR^+^/HER2^+^), HER2-positive and triple-negative (HR^-^/HER2^-^). Triple-negative breast cancer (TNBC) tends to be more aggressive than other types of breast cancer and shows the poorest prognosis^3^, while it has no apparent drug targets. Currently, the most efficient and widely used therapy for TNBC is the systemic chemotherapy, such as anthracyclines and taxanes. However in patients with TNBC, chemotherapy is associated with a high risk of recurrence and tumors often acquire drug resistance after relapse. Therefore, there is an urgent agenda to develop new targeted therapy options for TNBC.

**Figure 1:**
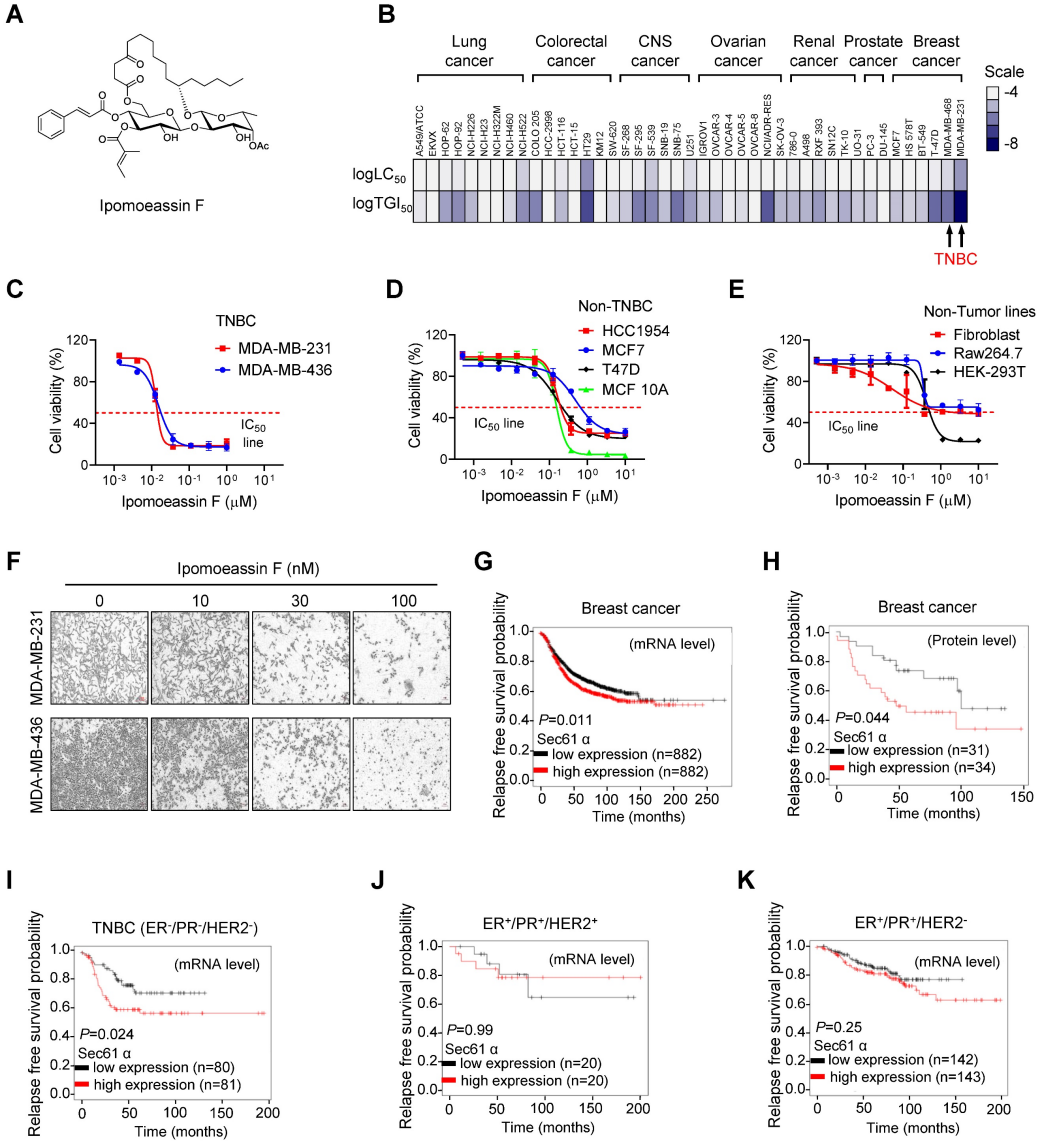
** Selective inhibition of TNBC cell growth by ipomoeassin F. (A)** The chemical structure of ipomoeassin F. **(B)** Heatmap analysis of logTGI_50_ and logLC_50_ of ipomoeassin F in different types of cancer cells obtained from NCI60 drug sensitivity profile (National Cancer Institute Developmental Therapeutics Program). The two TNBC cell lines, MDA-MB-231 and MDA-MB-468, are highlighted. **(C-E)** Effect of ipomoeassin F on cell viability of two TNBC lines **(C)**, four non-TNBC lines **(D)** and three non-tumor lines **(E)** were analyzed. The cells were treated with ipomoeassin F for 5 days and an AlamarBlue assay was conducted to detect cell viability. **(F)** The representative images of MDA-MB-231 and MDA-MB-436 cells after treated with the indicated concentration of ipomoeassin F for 5 days. Scale bar = 20 μm. **(G-K)** The clinical association between sec61α expression level and patients' relapse-free survival probability using Kaplan-Meier plot. Differences in the probability of relapse-free survival probability between the high and low sec61α mRNA expression groups **(G)** and protein expression groups **(H)** in all types of breast cancer are shown. Differences in the relapse-free survival probability between the high and low sec61α mRNA expression groups in the ER^-^/PR^-^/HER2^-^ subtype (TNBC) **(I)**, ER^+^/PR^+^/HER2^+^ subtype **(J)** and ER^+^/PR^+^/HER2^-^ subtype **(K)** are shown. The log-rank test was used for statistical analysis. * *P* < 0.05 represents a significant difference between two analyzed groups.

Ipomoeassin F (Fig. 1A) is a natural product belonging to the ipomoeassin family of glycoresins isolated from *Ipomoea squamosa*^4^, ^5^. The ipomoeassin family of glycoresins have shown anticancer effects and potential antiviral activity^6^, ^7^. Ipomoeassin F is the most potent member of the family with IC_50_ values of low nanomolar concentrations^7^, ^8^. With an effort to identify molecular target of this natural product, Zong et al., recently discovered Sec61α, the pore-forming subunit of the endoplasmic reticulum (ER) translocon complex, as the direct binding target of ipomoeassin F^9^. The ER translocon complex plays an important role in transporting nascent polypeptides with a targeting signal peptide into the ER lumen from the cytosol^10^. It is composed of Sec (secretory) proteins that form a large protein complex with a transmembrane channel across the ER membrane. Sec61α that forms the central channel is a subunit of the hetero-trimeric protein complex called Sec61^11^. The binding of ipomoeassin F to Sec61α strongly inhibited the protein translocation into the ER, ER membrane insertion of the type II membrane protein and the protein secretory pathway^9^. Although the effect of ipomoeassin F on the Sec61α-containing ER translocon has been elucidated, its effects on the ER at the proteome level and anticancer activity have not been systematically investigated.

In this study, we show that ipomoeassin F is a selective inhibitor of TNBC cell growth. Through a quantitative proteomic approach, we establish that ipomoeassin F inhibits the levels of a group of proteins functioning in the ER, including the protein disulfide isomerases (PDIs) PDIA6 and PDIA4, in TNBC cells. Further studies demonstrate that ipomoeassin F induces ER stress and autophagy in TNBC cells by inhibiting ER translocation of PDIA6 and PDIA4, ER molecular chaperons critical for protein folding and quality control. This study highlights that ipomoeassin F is a potential drug lead for the treatment of TNBC and provides the first pharmacological mechanism of the drug action.

## Materials and Methods

### Cell culture

MDA-MB-231, MDA-MB-436, MCF7, Raw264.7, Fibroblast-AG07725 and HEK-293T were cultured in DMEM medium. HCC1954 and T47D were cultured in RPMI 1640 medium and MCF 10A cells were cultured in DMEM/F12 (Invitrogen) at 37 ℃ with 5% CO_2_. Fibroblast-AG07725 was a kind gift of Prof. Wakam Chang at the University of Macau and cultured in medium supplemented with 15% fetal bovine serum (FBS) and 1% penicillin/streptomycin (P/S). MCF 10A was a kind gift of Prof. Henry Kwok at the University of Macau and cultured in medium supplemented with 5% horse serum, 20 ng/mL EGF, 10 μg/mL insulin, 1 ng/mL cholera toxin, 0.5 μg/mL hydrocortisone and 1% penicillin/streptomycin (P/S). Other cells were obtained from American Type Culture Collection (ATCC) and cultured in medium supplemented with 10% FBS and 1% P/S. All medium and supplements were purchased from Life Technologies (USA).

### Compounds, plasmids and antibodies

Ipomoeassin F was chemically synthesized as described previously^12^, ^13^. PDI inhibitor CCF 642 (346640-08-2) was purchased from Tocris Bioscience (Shanghai, China). Thioflavin T (Th T) and Cycloheximide (CHX) were obtained from Sigma-Aldrich (USA). mRFP-GFP-MAP1LC3B plasmid was used as previously described^14^. All antibody information is indicated as following: PDIA6 (#18233-1-AP, Proteintech), Calnexin (#10427-2-AP, Proteintech), GRP78/Bip (#11587-1-AP, Proteintech), ERp72 (PDIA4) (#2798, CST), eIF2α (#5324, CST), pho-eIF2α (#3597, CST), cleaved-caspase3 (#9664, CST), LC3B (#2775, CST), GAPDH (sc-3650620, Santa Cruz), α-Tubulin (sc-5286, Santa Cruz), anti-mouse linked with HRP (sc-2005, Santa Cruz), anti-rabbit IgG linked with HRP (sc-2004, Santa Cruz), Alexa Fluor 488 donkey anti-mouse antibody (#A21202, Thermo Fisher), and Alexa Fluor 594 donkey anti-rabbit antibody (#A21207, Thermo Fisher). Details for the use of antibodies can be found in Supplementary Table S2.

### Proteome labeling and liquid chromatography-mass spectrometry LC-MS/MS analysis

Stable isotope labeling by amino acids in cell culture (SILAC)-based quantitative proteomic method^15^, ^16^ was applied to identify and quantify the proteins that were differentially expressed in MDA-MB-231 cells treated with or without ipomoeassin F as described previously^17^-^19^. Briefly, the proteome of the MDA-MB-231 cells was isotopically labeled for two weeks by growing the cells in media containing either unlabeled arginine and lysine ('light'), ^13^C_6_-arginine and ^2^H_4_-lysine ('medium') or ^13^C_6_^15^N_4_-arginine and ^13^C_6_^15^N_2_-lysine ('heavy') supplemented with 10% dialyzed FBS. The isotopically labeled cells were then treated with 18 nM ipomoeassin F for 0 h (in 'light' cells), 12 h (in 'medium' cells) or 16 h (in 'heavy' cells). Cells were harvested and lysed in a RIPA buffer (25 mM Tris-HCl, pH 7.5, 150 mM NaCl, 1% NP-40, 1% sodium deoxycholate, 0.1% SDS, 1x protease inhibitors (Roche Applied Science, Mannheim, Germany), and phosphatase inhibitors (10 mM NaF, 10 mM β-glycerophosphate, and 1 mM Na_3_VO_4_)), and sonicated on ice (Branson Digital Sonifier 450, Branson Ultrasonics Co., CT; 15% power, 10 x 15 second pulses with 20 second intervals between pulses). The lysate was centrifuged at 21,000 × g for 15 min at 4 °C, and the total protein (supernatant) concentration was determined by an RC DC Kit (Bio-Rad, Hercules, CA).

In-gel digestion, database search, and quantification of the LC-MS/MS data were performed as described previously^18^, ^20^. Specifically, equal amounts of the 'light', 'medium' and 'heavy' total protein were mixed, and the mixed protein was fractionated by a 12% SDS-PAGE. Each protein lane (the entire lane) of the Coomassie brilliant blue-stained gel was cut into 12 slices, and the gel slices were subjected to in-gel digestion with trypsin (Promega, Madison, WI)^17^, ^21^. The resulting peptides were analyzed by LC-MS/MS using an Orbitrap Fusion mass spectrometer (Thermo Fisher, San Jose, CA) operated in a data-dependent mode for tandem MS as described previously^22^, ^23^. Raw data from the LC-MS/MS analysis were processed by MaxQuant (version 1.6.1.0) with the built-in search engine Andromeda, and searched against a target-decoy human SwissProt protein database (October 2017) retrieved from UniProt (www.uniprot.org)^24^-^27^. The false discovery rates (FDRs) for peptide and protein identification were both set to 1%. The MS error tolerance was set to 4.5 ppm, and the MS/MS error tolerance was set to 0.5 Da. The minimum required peptide length was set to 7 amino acids, and a maximum of 2 missed cleavages was allowed. The variable modifications of ^13^C_6_-arginine, ^2^H_4_-lysine, ^13^C_6_^15^N_4_-arginine, ^13^C_6_^15^N_2_-lysine, oxidetion of methionine and protein N-terminal acetylation, and the fixed modification of cysteine carbamidomethylation were included. SILAC ratios (heavy/light and medium/light ratios) were calculated using the unique and razor peptides with a minimum ratio count of 2^25^. The proteins that matched to the reverse database, identified only by site or single peptide, and the common contaminants were discarded. The normalized SILAC ratios (heavy/light and medium/light ratios), which were normalized based on the assumption that the large population of proteins and their respective peptides do not exhibit substantial changes between any two conditions were used to represent protein expression changes^27^. Differences in protein abundance between different treatment durations of ipomoeassin F were reflected by the differences in MS signal intensity between heavy, medium and light peptides, and represented by the SILAC ratio (heavy/light and medium/light ratios).

### Alarma Blue cell viability assay

Cells were seeded in 96-well plates and cultured with the indicated dose of ipomoeassin F for 5 days. Alarma Blue reagent containing 0.25 mg/mL resazurin sodium salt powder (Sigma-Aldrich) in phosphate buffered saline (PBS) was added to the medium at 10% (v/v) to examine cell viability by measuring fluorescence at ex560/em590. The IC_50_ for the individual cell lines was analyzed with GraphPad Prism 8.0.

### siRNA transfection

PDIA4 and PDIA6 siRNA were synthesized by Integrated DNA Technologies and transfected with Lipofectamine RNAiMAX reagent (Thermo Fisher Scientific) in 12-well plates. For each well, 2.5 μL 20 μM siRNA and 0.8 μL transfection reagent were mixed with 200 μL Opti-MEM medium, and the mixture was incubated at room temperature (RT) for 20 min and dropped into well, followed by seeding 800 μL suspension containing 2 × 10^4^ cells and growth in a 37 ℃, 5% CO_2_ incubator for 48 h. The siRNAs sequence can be found in Supplementary Table S3.

### Reverse transcription and quantitative real-time PCR (RT-qPCR)

Total RNA of cells was isolated with a extraction buffer (10 mM Tris pH 7.4, 0.25% Igepal CA-630, 150 mM NaCl)^28^. Six-well plate confluent of cells was washed twice with PBS and 300 μL extraction buffer was added to each well, followed by incubation for 5-10 min at RT. The extract was harvested by pipetting the solution without disturbing the cell precipitation, and 3 μL lysis was reverse transcribed by a High-Capacity cDNA Reverse Transcription Kit (Thermo Fisher Scientific). qPCR was done with iTaq Universal SYBR Green Supermix (Bio-Rad) in CFX96 Real-Time PCR System (Bio-Rad, Hercules, CA). The primer sequence used can be found in Supplementary Table S4.

### Western blotting

Whole-cell proteins were prepared using 2 × laemmli lysis buffer (62.5 mM Tris-HCl, pH 6.8, 1% SDS, 10% glycerol, 10% 2-mercaptoethanol, 0.005% bromophenol blue). Tumor tissue-proteins were extracted in RIPA buffer (50 mM Tris-HCL, pH 8.0, 0.1% SDS, 150 mM NaCl, 1% Triton X-100, 0.5% sodium deoxycholate, 1 × protease Inhibitor Cocktail), protein quantification was done by BCA Protein Assay Kit (Thermo Fisher Scientific). An equal amount of proteins from every sample were boiled and resolved with SDS-PAGE, and the separated proteins were transferred onto PVDF membrane, followed by blocking with 5% milk, incubating with primary antibody, HRP-linked secondary antibody in turn, finally visualized on a ChemiDoc MP imaging system (Bio-Rad, Hercules, CA).

### Immunofluorescence

Cells grown on 8-well Chambered Coverglass (Thermo Fisher Scientific) were fixed with 4% paraformaldehyde (PFA) at 37 °C for 30 min after 48 h treatment with ipomoeassin F. Permeabilization and blocking were then performed in 3% BSA supplemented with 0.1% Triton X-100 for 30 min at RT. After washing with PBS 3 times, cells were incubated with primary antibody and Alexa Fluor 488-conjugated-secondary antibody at 4 ℃ overnight and at RT for 1 h, respectively. Cells and the nuclei were stained and mounted with Fluoromount-GTM containing DAPI (Thermo Fisher Scientific) for 5 min and the immunofluorescence images were acquired by Zeiss LSM 710 Confocal microscope (Carl Zeiss, Thornwood, NY).

### Measurement of autophagic flux

Autophagic flux was measured in cells transfected with mRFP-GFP-MAP1LC3B plasmid. Cells grown on 8-well Chambered Coverglass (Thermo Fisher Scientific) were transfected with the mRFP-GFP-MAP1LC3B plasmid using the Lipofectamine 3000 reagent (Thermo Fisher Scientific) according to the manufacturer's protocol. After 24 h of transfection, cells were treated with an indicated compound for additional 24 h. The cells were then processed for immunofluorescence and observed under a confocal microscope for GFP and mRFP fluorescent images. The numbers of GFP and RFP dots per cell were counted and quantitated as early and late autophagosomes, respectively. In the merged images, the number of free red dots representing autolysosomes and the number of yellow dots representing autophagosomes were counted and quantified.

### Misfolded protein detection

Cells cultured in 8-well Chambered Coverglass or 96-well plates under 50 nM ipomoeassin F treated for 12 h were washed with PBS twice. Viable cells were fixed with 4% PFA at 37 ℃ for 30 min, then permeabilized and blocked in 3% BSA supplemented with 0.1% Triton X-100 for 30 min at RT. After washing with PBS 3 times, the cells were stained with 40 μM Th T for 30 min at 37 ℃. For coverglass, the nucleus was stained with DAPI and the immunofluorescence images were acquired by Zeiss LSM 710 Confocal microscope. For 96-well plates, cells were washed by PBS twice and the fluorescence signal was determined at ex440/em490.

### Mouse experiments

Twenty-four athymic nude mice (female, 8-weeks, six mice/group) were raised in specific pathogen-free conditions and subcutaneously injected with 2 × 10^6^ MDA-MB-231 cells into the right flanks. When the tumors grew to about 100 mm^3^, daily intraperitoneal injections of vehicle (sterile saline containing 5% dimethyl sulfoxide, 5% tween-80, and 5% polyethylene glycol-400) or ipomoeassin F (0.25, 0.5 and 1 mg/kg) in vehicle were performed for 7 days, followed by daily intraperitoneal injections of vehicle or ipomoeassin F (0.5, 0.75 and 1.5 mg/kg) in vehicle for additional 18 days according to its in vivo maximum tolerated dose^13^. Finally, the mice were sacrificed and the tumor samples were harvested for further analysis. The tumor volume was calculated by the modified ellipsoid formula (long axis × short axis^2^/2). All animal experiments were approved by the Animal Research Ethics Committee of the University of Macau and conducted according to ARRIVE guidelines^29^.

### Data analysis

All experiments were repeated at least three times. Statistical significance between the two groups was analyzed by the unpaired two-tailed Student's t-test with GraphPad Prism 8.0 software.

## Results

### Ipomoeassin F, a Sec61α inhibitor, is a selective inhibitor of TNBC cell growth

To investigate cancer type selectivity of ipomoeassin F, we analyzed the NCI-60 drug sensitivity profile that we reported previously for ipomoeassin F^30^. Based on the lethal concentration at 50% (LC_50_) and the tumor growth inhibitory concentration at 50% (TGI_50_) of ipomoeassin F on 43 human cancer cell lines, including lung cancer, colorectal cancer, central nervous system (CNS) cancer, ovarian cancer, renal cancer, prostate cancer and breast cancer cells, we found that TNBC cell lines were amongst the most sensitive cell types to ipomoeassin F (Fig. 1B, Supplementary Table S1). To further validate this result, we examined the inhibitory effect of ipomoeassin F on the cell growth in three groups of cell lines, including non-tumor origin lines (Raw264.7, Fibroblast-AG07725, and HEK-293T), breast epithelial and non-TNBC breast cancer lines (MCF10A, MCF7, HCC1954 and T47D) and TNBC lines (MDA-MB-436 and MDA-MB-231). As a result, non-tumor origin lines were least sensitive (average IC_50_ of 2.89 μM), non-TNBC lines were moderately sensitive (average IC_50_ of 237 nM) and TNBC lines were most sensitive (average IC_50_ of 20 nM) to ipomoeassin F (Fig. 1C-F).

Considering that Sec61α is the target protein of ipomoeassin F for its pharmacological activity^9^, we next analyzed clinical association between Sec61α level and breast cancer patients' survival. The gene expression-associated survival analysis of breast cancer patients with Kaplan-Meier Plotter (https://kmplot.com/analysis/) showed that the low level of Sec61α transcripts or protein expression is associated with longer relapse-free survival (Fig. 1G and H) and overall survival (Supplementary Fig. S1) of breast cancer patients. We then divided the breast cancer patients into three subgroups based on the molecular subtypes: ER^+^/PR^+^/HER2^-^ (hormone receptor-positive), ER^+^/PR^+^/HER2^+^ (hormone receptor and HER2-positive) and ER^-^/PR^-^/HER2^-^ (triple-negative), and analyzed each subgroup for the Sec61α-associated patients' survival. Surprisingly, only the TNBC subgroup (ER^-^/PR^-^/HER2^-^) showed a significantly longer relapse-free survival when Sec61α expression level is low (Fig 1I-K). Together, these data suggested a potential role of Sec61α in TNBC growth and patients' survival, and that ipomoeassin F has a potential to be a selective inhibitor of TNBC growth.

### Proteomic analysis reveals that ipomoeassin F reduces the levels of ER molecular chaperones and induces ER stress in TNBC cells

**Figure 2:**
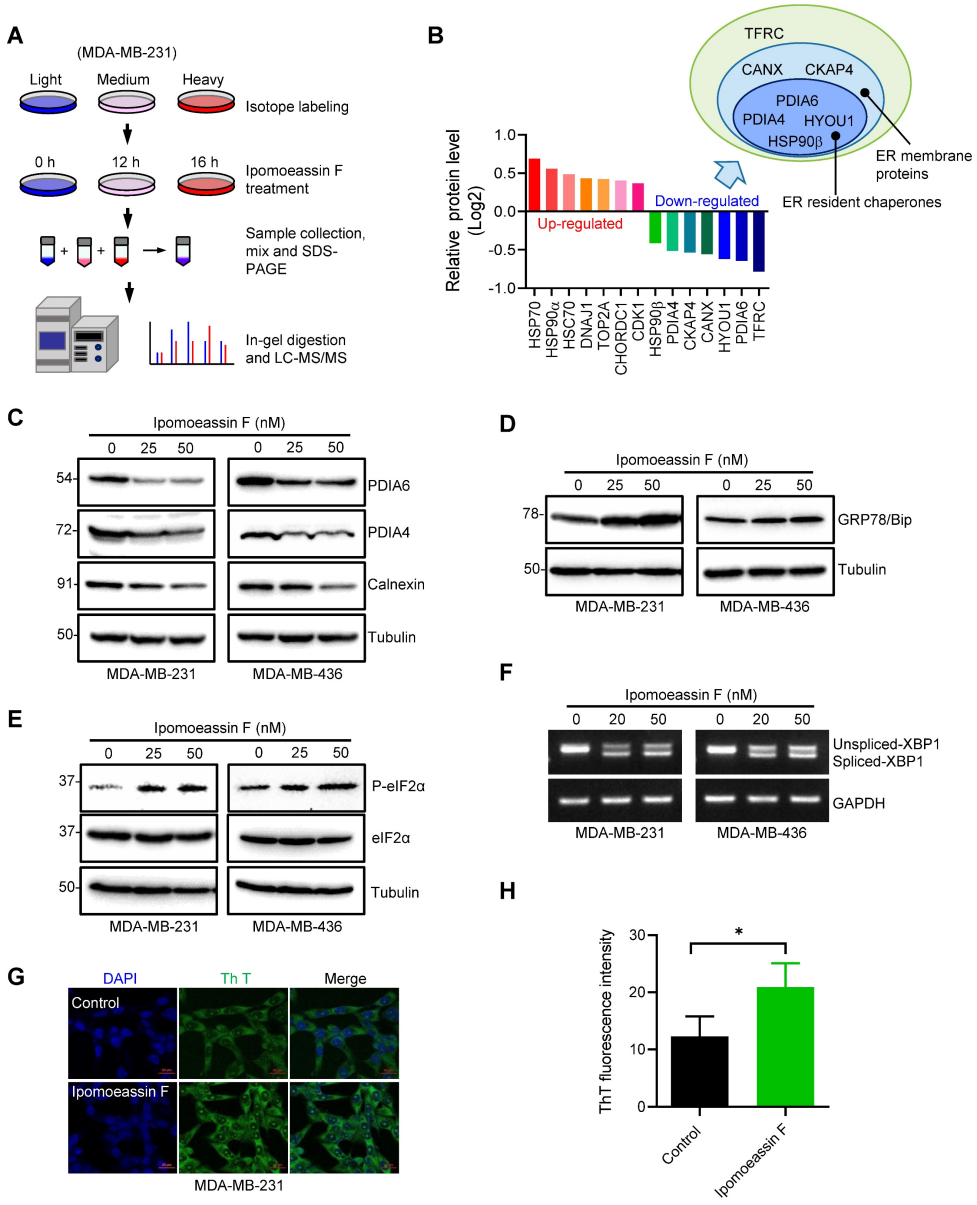
**Ipomoeassin F down-regulates PDIA6 and PDIA4 and induces ER stress in TNBC cells. (A)** A schematic illustration of the SILAC procedure to identify the differentially expressed proteins in MDA-MB-231 cells treated with ipomoeassin F. **(B)** Identification of the top 7 most reliably up- and down-regulated proteins by ipomoeassin F. As shown, most of the seven down-regulated proteins are localized in the ER. **(C)** Effect of ipomoeassin F on the protein levels of PDIA6, PDAI4 and calnexin in MDA-MB-231 and MDA-MB-436 was analyzed by Western blotting. Cells were treated with ipomoeassin F at 25 nM or 50 nM for 48 h. (**D**,** E)** The protein levels of GRP78, eIF2α and phosphorylated eIF2α (P-eIF2α) revealed by Western blotting in TNBC cells treated with the indicated concentrations of ipomoeassin F for 48 h. **(F)** TNBC cells were treated with 20 nM or 50 nM ipomoeassin F for 48 h, and PCR-amplified products of XBP1 cDNA were resolved by agarose gel electrophoresis. Tubulin or GAPDH was used as a loading control for Western blotting or PCR analysis, respectively. **(G)** Analysis of intracellular misfolded proteins with thioflavin T (Th T). MDA-MB-231 cells were treated with 50 nM ipomoeassin F for 12 h, and then stained with 40 μM Th T for 30 min at 37 ℃. The nuclei were stained with DAPI. Scale bar = 20 μm. **(H)** The fluorescence intensity of Th T from **(G)** is shown in a bar chart, and the difference between the two groups was analyzed by Student's t-test. *P < 0.05.

Sec61α inhibition by ipomoeassin F can cause disruption of protein transport into or across the ER membrane, and hence can lead to changes in the levels of a number of affected proteins due to misfolding or mislocalization. To detect the changes in protein levels globally, we conducted proteomic analyses of TNBC cells treated with ipomoeassin F. We used a stable isotope labeling by amino acids in cell culture (SILAC)-based quantitative proteomic method^15^, ^16^ to profile protein expression in the MDA-MB-231 cells treated with a fixed concentration of ipomoeassin F (18 nM) for different periods of time (0, 12, 16 h) (Fig. 2A). Among the identified and quantified proteins, we identified 14 of the most reliably up- or down-regulated proteins based on the SILAC ratios (which represent the relative expression between the ipomoeassin F-treated cells and untreated cells), the number of unique peptides and sequence coverage (Fig. 2B). Among the up-regulated proteins, 4 of them were heat shock proteins, including HSP70, HSP90α, HSC70 and DNAJ1 (HSP40), suggesting that ipomoeassin F caused a cellular phenotype that requires chaperone functions. Since we were more interested in the proteins whose level was reduced upon inhibition of the ER translocon by ipomoeassin F, we looked more closely at the 7 down-regulated proteins, including TFRC (transferrin receptor protein 1), PDIA6 (protein disulfide-isomerase A6), HYOU1 (hypoxia up-regulated protein 1, also called as GRP170), CANX (calnexin), CKAP4 (cytoskeleton-associated protein 4), PDIA4 (protein disulfide-isomerase A4) and HSP90β (also called as endoplasmin). Interestingly, majority of the down-regulated proteins are molecular chaperones that are localized in the ER, including 4 of ER resident chaperones (PDIA6, HYOU1, PDIA4 and HSP90β) and 2 of ER membrane proteins (CANX and CKAP4), suggesting that chaperones in the ER were highly sensitive to the Sec61α inhibitor treatment.

Given that PDIA6 and PDIA4 were amongst the top down-regulated ER resident chaperones upon the ipomoeassin F treatment, we selected the two proteins for mechanistic studies of ipomoeassin F in TNBC cells. PDIA6 and PDIA4 are ER molecular chaperones and are members of the protein disulfide-isomerase family of proteins whose function is critical for protein folding in the ER^31^-^33^. We first verified the proteomics results of the levels of PDIA6 and PDIA4 in MDA-MB-231 and MDA-MB-436 TNBC cells. Ipomoeassin F treatment reduced the protein levels of PDIA6 and PDIA4 in both TNBC cell lines (Fig. 2C). CANX (calnexin), another ER chaperones identified from the proteomics data was also down-regulated by ipomoeassin F (Fig. 2C). However, we did not observe the reduction in the mRNA levels of PDIA6 and PDIA4 in the same cell lines (Supplementary Fig. S2A and B), suggesting that ipomoeassin F reduces the levels of PDIA6 and PDIA4 via a post-translational mechanism.

Down-regulation of ER chaperone proteins could lead to accumulation of unfolded or misfolded proteins in ER, which in turn could cause ER stress and unfolded protein response (UPR). Indeed, TNBC cells treated with ipomoeassin F dose-dependently upregulated GRP78/Bip (Fig. 2D), an ER stress marker^34^. We then analyzed two UPR pathways, the PERK and IRE1 pathways, in TNBC cells treated with ipomoeassin F. Ipomoeassin F significantly increased the phosphorylated form of eIF2α, a down-stream marker for PERK signaling^35^ (Fig. 2E) and the spliced form of XBP1 mRNA, a downstream effector of the IRE1 signaling cascade^36^ (Fig. 2F) in both MDA-MB-231 and MDA-MB-436 TNBC cell lines. We further showed the accumulation of misfolded proteins in ipomoeassin F-treated cells using thioflavin T (Th T), a fluorescent dye widely used to visualize the level of amyloid fibril and the formation of misfolded protein aggregates in vitro and in vivo^37^. Ipomoeassin F significantly increased the fluorescence intensity of Th T in TNBC cells (Fig. 2G and H), implying that ipomoeassin F indeed induced misfolded protein accumulation. These results demonstrated that ipomoeassin F down-regulated the protein levels of ER molecular chaperones, including PDIA6 and PDIA4, leading to the induction of ER stress and UPR in TNBC cells.

### Inhibition of PDIA6 and PDIA4 promotes ER stress and UPR in TNBC cells

**Figure 3:**
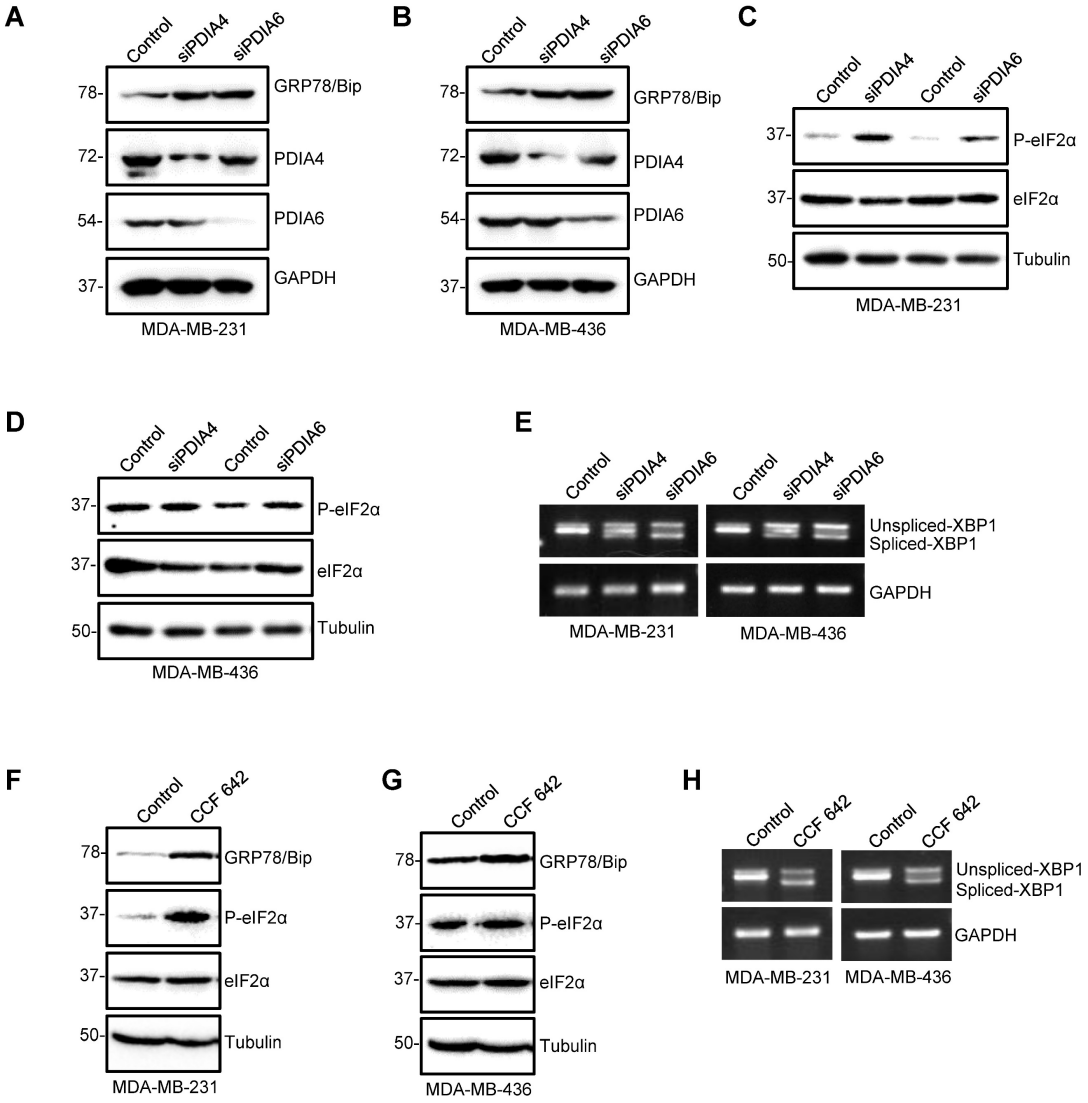
** Induction of ER stress and UPR by PDIA4 or PDIA6 inhibition in TNBC cells. (A-E)** Effects of PDIA4 and PDIA6 silencing on the expression of the key factors functioning in the ER stress and UPR signaling. After MDA-MB-231 and MDA-MB-436 cells were transfected with 50 nM PDIA4 or PDIA6 siRNAs for 48 h, the protein levels of PDIA4, PDIA6 and GRP78 **(A, B),** and P-eIF2α and eIF2α **(C, D)** were determined by western blotting, and the PCR-amplified XBP1 cDNA was analyzed by agarose gel electrophoresis **(E)**. **(F-H)** Effects of PDIA4 and PDIA6 function blocking on the expression of the key factors functioning in the ER stress and UPR signaling. After MDA-MB-231 and MDA-MB-436 cells were treated with 2.5 μM PDIs inhibitor CCF 642 for 48 h, the expression of PDIA4, PDIA6, GRP78, P-eIF2α and eIF2α was determined by Western blotting **(F, G)**, and the PCR-amplified XBP1 cDNA was analyzed by agarose gel electrophoresis **(H)**. Tubulin and GAPDH were used as the loading control.

To explore the role of PDIA6 and PDIA4 during the process of the observed ER stress induced by ipomoeassin F, we first tested the effect of the siRNA silencing of PDIA6 and PDIA4 on the induction of ER stress and UPR signaling in TNBC cells. The silencing of either PDIA6 or PDIA4 increased the level of GRP78/Bip (Fig. 3A and B), the level of phosphorylated eIF2α (Fig. 3C and D) and the level of the spliced form of XBP1 mRNA (Fig. 3E) in both MDA-MB-231 and MDA-MB-436 TNBC cell lines. The treatment of TNBC cells with CCF 642, a small molecule inhibitor of PDI, showed the same phenotypes as those for the PDIA6 or PDIA4-silenced cells, including the increase in the level of GRP78/Bip, phosphorylated eIF2α and the spliced form of XBP1 mRNA (Fig. 3F-H). These results indicated that the down-regulation of PDIA6 and PDIA4 could be the primary event that mediated the ipomoeassin F-induced ER stress and UPR.

### Inhibition of PDIA6 and PDIA4 increases autophagic flux in TNBC cells

**Figure 4:**
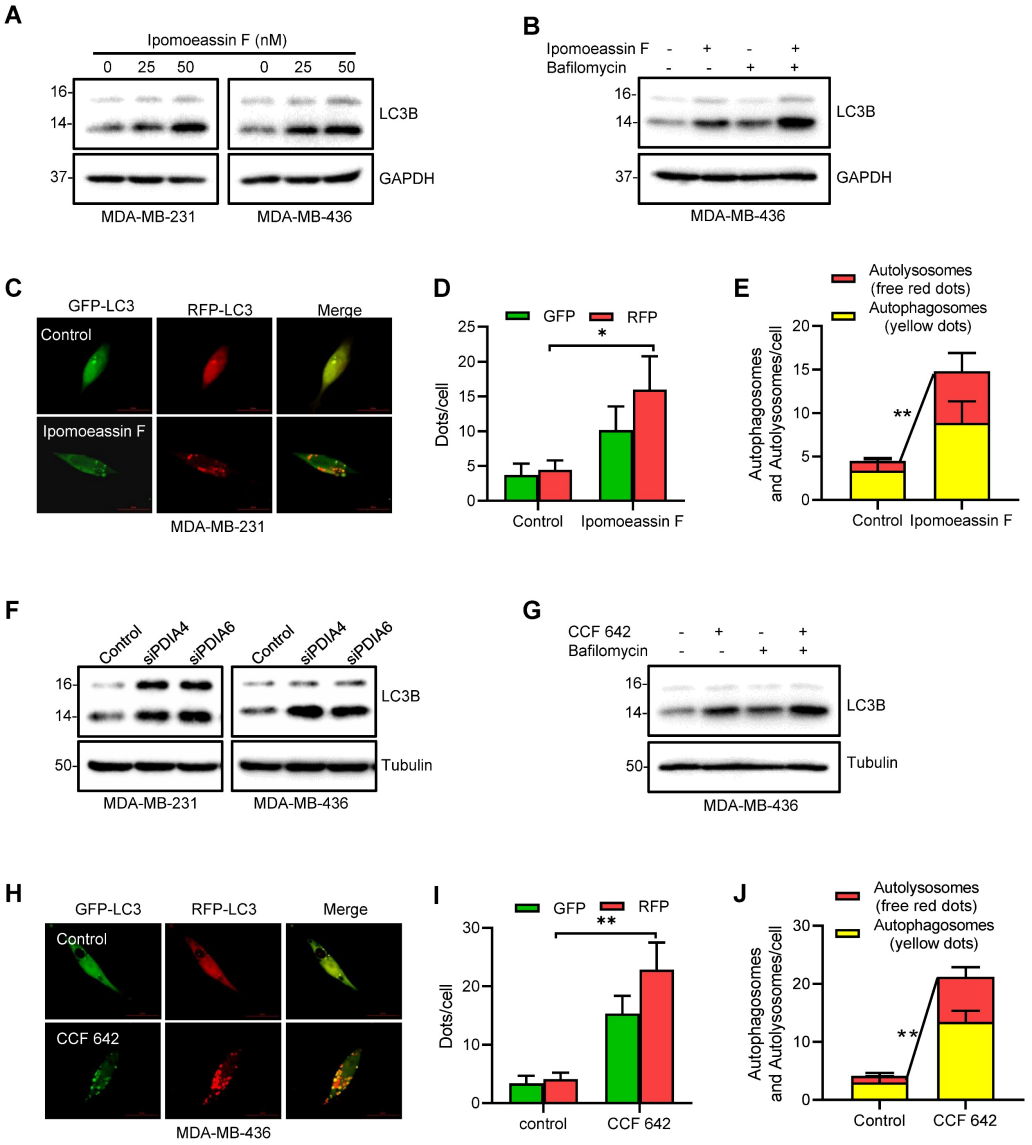
** Activation of autophagy in TNBC cells by ipomoeassin F and protein disulfide isomerase inhibition. (A)** Effect of ipomoeassin F on the protein levels of LC3B. MDA-MB-231 and MDA-MB-436 cells were treated with 25 nM or 50 nM ipomoeassin F for 48 h, and the protein levels of LC3B were determined by Western blotting. **(B)** Effect of ipomoeassin F and bafilomycin on the protein levels of LC3B. MDA-MB-436 cells were treated with 50 nM ipomoeassin F for 48 h with or without 100 nM bafilomycin for 2 h, and the levels of LC3B proteins were then analyzed by Western blotting. The upper band represents the LC3-I and the lower band represents LC3-II isoform. **(C-E)** Effect of ipomoeassin F on induction of autophagy. Twenty-four hours after transfection of MDA-MB-231 cells with the mRFP-GFP-MAP1LC3B-expressing plasmid, the transfected cells were incubated with 50 nM ipomoeassin F for 24 h. The fluorescent images **(C)** were then captured, and the numbers of GFP and RFP dots per cell were counted **(D).** Free red dots (representing autolysosomes) and yellow dots (representing autophagosomes) were also counted in the merged images and quantified **(E)**, scale bar = 20 μm. **f** Effect of PDIA4 or PDIA6 knockdown on LC3B protein levels in MDA-MB-231 and MDA-MB-436 cells revealed by Western blotting. **(G)** Effect of CCF 642 and bafilomycin on the protein levels of LC3B. MDA-MB-436 cells were treated with 50 nM ipomoeassin F for 48 h with or without 100 nM bafilomycin for 2 h, and the levels of LC3B proteins were then analyzed by Western blotting. **(H-J)** Effect of CCF 642 on induction of autophagy. Twenty-four hours after transfection of MDA-MB-231 cells with the mRFP-GFP-MAP1LC3B-expressing plasmid, the transfected cells were incubated with 2.5 μM CCF 642 for 24 h. The fluorescent images **(H)** were then captured, and the numbers of GFP and RFP dots per cell were counted **(I).** Free red dots (representing autolysosomes) and yellow dots (representing autophagosomes) were also counted in the merged images and quantified **(J)**, scale bar = 20 μm. The statistical difference between two indicated groups was analyzed by Student's t-test. *P < 0.05, **P < 0.01.

ER stress often leads to the induction of autophagy, a process of the self-clearance of damaged organelles and misfolded proteins in cells as a cellular protective mechanism. However, when the protein and organelle turnover overwhelms the capacity of the cell in the prolonged ER stress, cell death will be triggered. The balance between autophagy and apoptotic cell death controls the cell fate after ER stress^38^. To determine the cell fate after ER stress, we first examined autophagy in TNBC cells treated with ipomoeassin F. Ipomoeassin F treatment dose-dependently increased the LC3-II conversion in both MDA-MB-231 and MDA-MB-436 cells (Fig. 4A). The co-treatment of TNBC cells with bafilomycin A, a lysosomal V-ATPase inhibitor, further increased the ipomoeassin F-induced LC3-II conversion, suggesting that ipomoeassin F increased the autophagic flux (Fig. 4B). We further analyzed the autophagic flux in TNBC cells treated with ipomoeassin F using a double-tagged (RFP-GFP)-LC3. This tandem fluorescent tagged LC3 can visualize the transition of neutral autophagosomes to acidic autolysosomes based on the different pH stability of RFP and GFP. RFP is relatively stable in acidic lysosomes, while GFP is unstable in the same condition. Therefore, autophagosomes will be stained as yellow dots (RFP+GFP), whereas autolysosomes will be stained as red dots (RFP only). The treatment of TNBC cell with ipomoeassin F significantly increased the numbers of GFP and RFP dots (Fig. 4C and D). As shown in the merged images, ipomoeassin F also significantly increased both autophagosomes (yellow dots) and autolysosomes (red dots) (Fig. 4C and E), suggesting that ipomoeassin F increased autophagic flux.

We then examined whether silencing of PDIA6/PDIA4 or inhibition of their protein disulfide isomerase activities could recapitulate the autophagy-promoting effect of ipomoeassin F. Silencing of either PDIA6 or PDIA4 significantly increased the LC3-II conversion in both MDA-MB-231 and MDA-MB-436 cells (Fig. 4F). Likewise, the PDI inhibitor CCF 642 increased the LC3-II conversion in TNBC cells and the LC3-II conversion was further increased by co-treatment with bafilomycin A (Fig. 4G). CCF 642 also increased both autophagosomes and autolysosomes in TNBC cells (Fig. 4H-J). These results suggested that ipomoeassin F induced ER stress, UPR and autophagy by suppressing PDIA6 and PDIA4 levels in TNBC cells.

**Figure 5:**
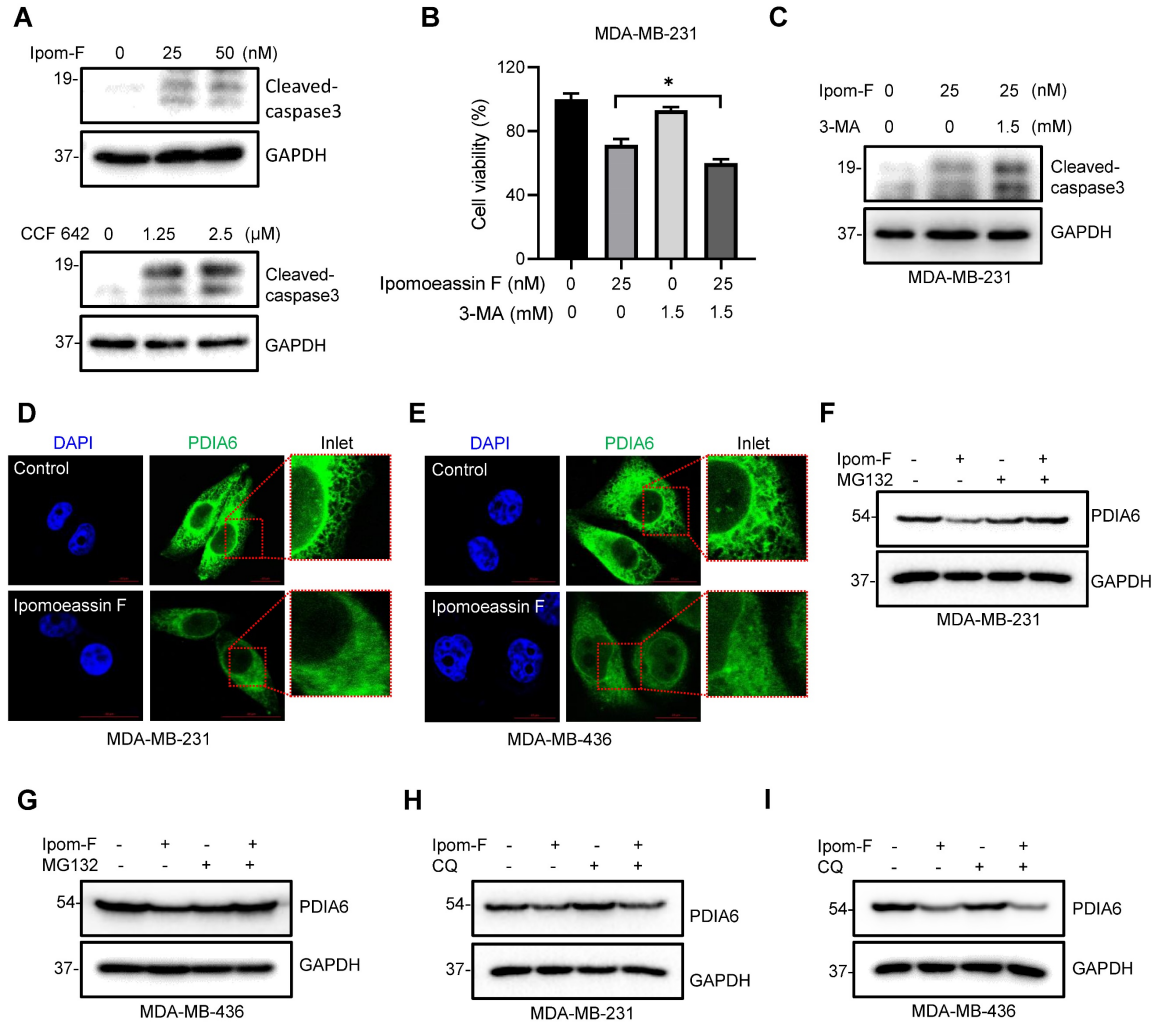
** Ipomoeassin F blocks PDIA6 translocation into ER and promotes its degradation in TNBC cells. (A)** Effects of ipomoeassin F and CCF 642 on caspase 3 cleavage in MDA-MB-231 cells. MDA-MB-231 cells were treated with the indicated concentrations of ipomoeassin F (Ipom-F) and CCF 642 for 48 h, and the level of cleaved-caspase 3 protein was analyzed by Western blotting. **(B, C)** Effect of 3-methyladenine (3-MA) co-treatment with ipomoeassin F on the viability **(B)** and caspase 3 cleavage **(C)** in TNBC cells. MDA-MB-231 cells were treated with 25 nM ipomoeassin F with or without 1.5 mM 3-MA for 48 h. The cell viability was analyzed by Alarma blue assay **(B)** and the level of cleaved-caspase 3 protein was analyzed by Western blotting **(C)**.** (D, E)** Ipomoeassin F inhibits ER translocation of PDIA6. MDA-MB-231 **(D)** and MDA-MB-436 **(E)** cells were treated with 50 nM ipomoeassin F for 16 h, the cells were stained with DAPI and fluorescence-labeled anti-PDIA6 antibody, and the immunofluorescence images were acquired. Inlet images (red square) were enlarged and shown at the right to each panel, scale bar = 20 μm. **(F, G)** Effect of MG132 on the ipomoeassin F-induced reduction of PDIA6 protein level. MDA-MB-231** (F)** and MDA-MB-436 **(G)** cells were treated with 50 nM ipomoeassin F for 24 h with or without 3 μM MG132 for 12 h, and the level of PDIA6 protein was analyzed by Western blotting. **(H, I)** Effect of chloroquine on the ipomoeassin F-induced reduction of PDIA6 protein level. MDA-MB-231 **(H)** and MDA-MB-436 **(I)** cells were treated with 50 nM ipomoeassin F for 24 h with or without 100 μM chloroquine for 12 h, and the level of PDIA6 protein was analyzed by Western blotting.

Cellular ER stress induced by the accumulation of misfolded proteins will trigger cellular protective mechanisms, including UPR and autophagy that can help clear misfolded proteins and restore ER homeostasis. However, prolonged ER stress that crosses the cellular clearance threshold will trigger apoptotic cell death. TNBC cells treated with ipomoeassin F or the PDI inhibitor CCF 642 showed an increase in the level of cleaved caspase-3, an indicative of apoptotic cell death triggered by these compounds (Fig. 5A). Treatment of TNBC cells with the autophagy inhibitor 3-methyladenine (3-MA) further potentiated the apoptotic cell death induced by ipomoeassin F (Fig. 5B and C). These data indicated that ipomoeassin F induced a lethal ER stress that overwhelmed the cellular clearance capacity in TNBC cells and that inhibition of the protective autophagy could potentiate the ipomoeassin F-induced cell death.

### Ipomoeassin F inhibits ER translocation of PDIA6 and promotes its degradation

To investigate the underlying mechanism of how ipomoeassin F downregulates the PDIA6 protein level, we performed an immunofluorescent staining of PDIA6 in TNBC cells treated with ipomoeassin F. In control MDA-MB-231 cells, PDIA6 staining showed a standard ER network structure in the cytosolic area, indicating its localization in the ER lumen (Fig. 5D). However in the cells treated with ipomoeassin F, PDIA6 staining was much weaker than in control cells and its localization was rather dispersed throughout the cytoplasm. A very similar phenotype was observed in MDA-MB-436 cells treated with ipomoeassin F (Fig. 5E), suggesting that ipomoeassin F blocked the translocation of PDIA6 into ER lumen and reduced its protein level.

Proteins that fail to undergo ER quality control pathways will be recognized and targeted for destruction in the cytosol by the ubiquitin-proteasome system (UPS). To examine whether the PDIA6 protein was destabilized by UPS, we tested the effect of the proteasome inhibitor MG132 on ipomoeassin F-mediated destabilization of PDIA6. The pretreatment of TNBC cells with MG132 significantly rescued ipomoeassin F-mediated reduction of PDIA6 (Fig. 5F and G), demonstrating that PDIA6 underwent UPS-mediated degradation upon ipomoeassin F treatment. The autophagy inhibitor chloroquine, however, could not rescue the ipomoeassin F-mediated destabilization of PDIA6 (Fig. 5H and I), excluding the possibility of the autophagy-mediated lysosomal degradation of PDIA6. Together these results suggested that the ER translocon inhibitor ipomoeassin F blocked the translocation of PDIA6 into the ER and promoted its degradation.

### Ipomoeassin F inhibits TNBC xenograft tumor growth in vivo

**Figure 6:**
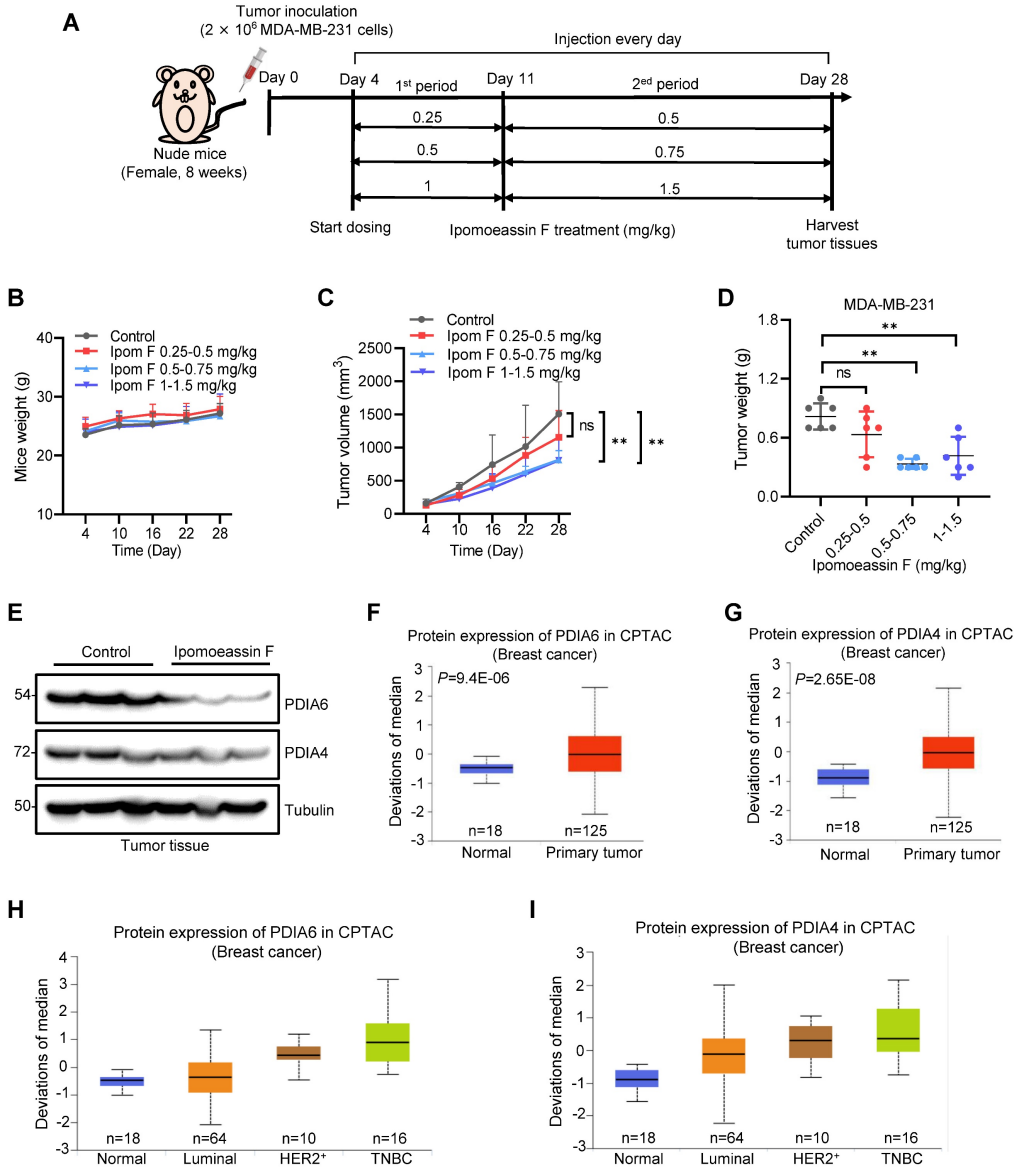
** In vivo antitumor activity of ipomoeassin F against TNBC tumor xenografts. (A)** Schematic illustration of the mouse tumor xenograft experiment and dosing schedule for ipomoeassin F. Mice bearing MDA-MB-231 tumors were divided into 4 dosing groups (n=6/group), including control and three dosing groups (0.25-0.5 mg/kg group, 0.5-0.75 mg/kg group, and 1-1.5 mg/kg group) of ipomoeassin F. Mice were treated with ipomoeassin F daily via i.p. injection for 7 days (1^st^ period with a low dose) and then 18 days (2^nd^ period with a high dose) successively. **(B)** The measurement of the body weights of the mice in each group. **(C)** The measurement of the tumor volumes in each group. **(D)** The measurement of the tumor wet weights in each group after being harvested from the mice. Data are mean ± SD of each group, and the statistical differences between control and ipomoeassin F treatment groups were analyzed by Student's t-test. ns denotes not significant. **P < 0.01 between two indicated groups. **(E)** Analysis of PDIA6 and PDIA4 proteins levels in tumor tissues by Western blotting. Three randomly selected tumors from the control group and the 1-1.5 mg/kg group were analyzed as the representative tumor samples from each group. **(F-I)** Clinical data analysis of PDIA6 and PDIA4 levels in breast cancer patients' samples downloaded from the Clinical Proteomic Tumor Analysis Consortium (CPTAC) database. The PDIA6 **(F)** and PIDA4 **(G)** protein levels in normal breast tissues and primary breast tumor tissues are shown. The levels of PDIA6 **(H)** and PDIA4 **(I)** proteins in different breast tumor subtypes are shown. The log-rank test was used for statistical analysis, *P* < 0.05 represents statistically significant.

To assess the therapeutic effects of ipomoeassin F against TNBC in vivo, we conducted tumor xenograft experiments with MDA-MB-231 TNBC TNBC cells in athymic nude mice. Since ipomoeassin F is a highly potent compound with a low maximum tolerated dose (MTD: ~3 mg/kg)^13^, we designed a two-dose escalation scheme for mice dosing (Fig. 6A). Under this dose regimen, all mice were healthy without showing any signs of toxicity of ipomoeassin F (Fig. 6B). While there was no significant reduction in tumor volume and wet weight in the low dose group (0.25-0.5 mg/kg) of ipomoeassin F, the medium dose (0.5-0.75 mg/kg) and the high dose (1-1.5 mg/kg) groups of ipomoeassin F resulted in marked reductions in tumor volumes and wet weights (Fig. 6C and D). Western blot analysis of the tumor samples showed that ipomoeassin F significantly decreased the levels of PDAI6 and PDIA4 proteins (Fig. 6E), demonstrating the blocking effect of ipomoeassin F on PDIA6 and PDIA4 in vivo.

We lastly analyzed the clinical significance of the levels of PDIA6 and PDIA4 in breast cancer using the publicly available Clinical Proteomic Tumor Analysis Consortium (CPTAC, http://ualcan.path.uab.edu/cgi-bin/CPTAC) database. Both PDIA6 and PDIA4 protein levels were notably upregulated in primary breast cancers, compared to normal adjacent breast tissues (Fig. 6F and G). Moreover, the analysis of breast cancer subtypes showed that TNBC tumors express the highest level of PDIA6 and PDIA4 proteins among all the subtypes examined (Fig. 6H and I). These results suggested that PDIA6 and PDIA4 play important roles in TNBC survival and progression, and supported our notion that these ER molecular chaperones could be therapeutic targets for TNBC treatment.

## Discussion

**Figure 7:**
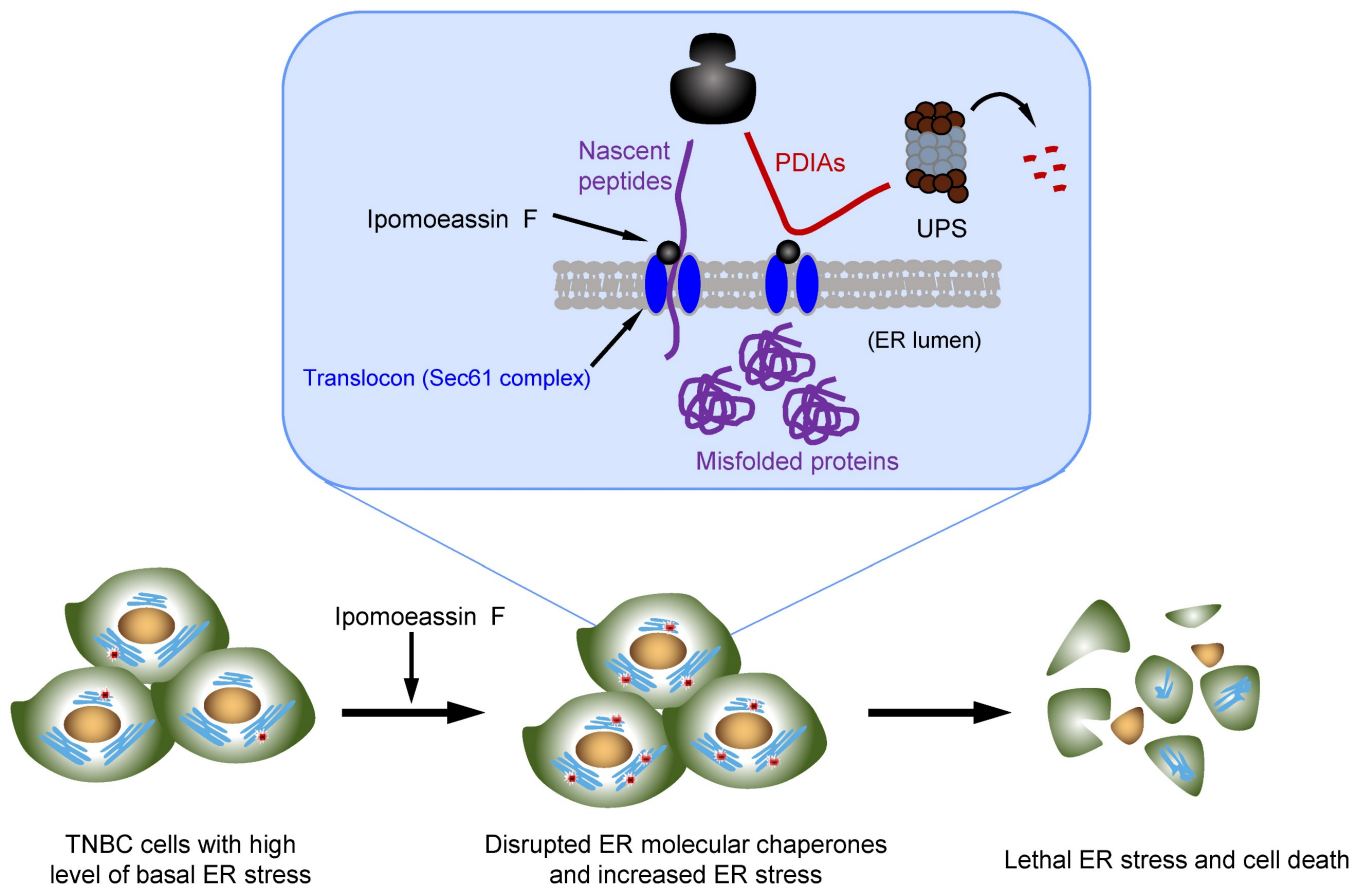
** Model of the ipomoeassin F-induced TNBC cell death.** Ipomoeassin F blocks the Sec61 complex function and inhibits the ER translocation of ipomoeassin F-sensitive ER molecular chaperones, including PDIA6 and PDIA4 in TNBC cells. PDIA6 and PDIA4 undergo ubiquitin-proteasome system (UPS)-mediated degradation. Low level of ER molecular chaperones in the ER lumen causes the accumulation of misfolded proteins, inducing lethal ER stress in TNBC cells where a high level of basal ER stress is present. Unresolved ER stress leads to TNBC cell death.

Natural products consist of various chemical entities that play important roles in regulating biological activities. Therefore, they have been attractive materials and leading molecules in the field of drug development, especially in anticancer drug development^39^. To date, numerous natural products with anticancer activities have been discovered. However, their complex chemical structure and the unspecified mechanism limit the clinical application of the natural products. Ipomoeassin F remained as one such natural product without a clear functional mechanism for its potent anticancer activity. As a part of effort to elucidate the anticancer mechanism of ipomoeassin F, our group recently identified Sec61α, a component of ER translocon complex, as the molecular target of ipomoeassin F in mammalian cells^9^. In the present study, we show that ipomoeassin F is a selective inhibitor of TNBC growth by inducing ER stress, UPR signaling and autophagy. Our proteomics and subsequent functional studies reveal that ipomoeassin F blocks ER translocation of the ER molecular chaperones, PDIA6 and PDIA4, and destabilizes them, causing the accumulation of cellular misfolded proteins and ER stress (Fig. 7).

The effect of ipomoeassin F on PDIA6 and PDIA4 is likely originated from its ability to inhibit the ER translocon protein Sec61α. Sec61α is the largest subunit of the heterotrimeric Sec61 complex that further interacts with the Sec62/63 complex, forming a protein conducting channel in the ER membrane^40^. This ER translocon complex allows translocation of nascent polypeptides into the ER lumen. Our recent in vitro studies suggest that some Sec61 client proteins are particularly sensitive to ipomoeassin F for their ER translocation. PDIs were alluded as one type of such ipomoeassin F-sensitive Sec61 client proteins^41^. Consistent with this previous report, our present study validates that PDIA6 and PDIA4 are the major ipomoeassin F-sensitive Sec61 client proteins, whose ER translocation is blocked, and protein stability is significantly reduced by ipomoeassin F. Moreover, we demonstrated that PDIA6 and PDIA4 silencing or their pharmacological inhibition was sufficient to recapitulate the ipomoeassin F-treated cell phenotypes, such as ER stress, UPR induction and autophagy activation. These results imply that the ER molecular chaperones PDIA6 and PDIA4 are among the major cellular targets through which ipomoeassin F exerts its TNBC inhibition activity.

The ER is the major organelle that serves many functions, including protein synthesis, the folding of proteins and the transport of newly synthesized proteins to the Golgi apparatus. ER molecular chaperones, such as PDIs, play a crucial role in the correct folding and quality control of ER translocated proteins^42^. Abnormal regulation of such chaperones could lead to the accumulation of misfolded proteins, disrupting ER homeostasis and inducing ER stress. Through a SILAC-based quantitative proteomic approach, we identified PDIA6 and PDIA4 as the primary chaperones that were targeted by the Sec61α inhibitor ipomoeassin F to induce ER stress in TNBC cells. We further found that the expression levels of PDIA6 and PDIA4 were higher in breast cancer tissues compared to normal breast tissues, with the highest level observed in the TNBC subtype, suggesting that PDIA6 and PDIA4 could serve as prognostic markers and therapeutic targets for TNBC.

Our study showed that ipomoeassin F is a selective inhibitor for TNBC cells. It is, however, unclear why ipomoeassin F showed such a selectivity toward TNBC. Our meta-analysis of the clinical data showed that Sec61α high expression was negatively correlated with the survival rates of the TNBC patients, while there was no significant correlation between Sec61α level and the survival rates for other types of breast cancer, such as HR-positive and HER2-positive breast cancer. This data suggested that Sec61α-containing ER translocon complex and ER stress triggered by ipomoeassin F-mediated inhibition of ER translocon plays a distinct role in controlling TNBC survival and growth. In recent years, it has been widely recognized that cancer cells have higher levels of persistent ER stress than normal cells due to their diverse genetic and metabolic abnormalities and altered microenvironments, such as accumulated reactive oxygen species, hypoxia and low pH^43^. The non-lethal, sustained ER stress responses help restore ER homeostasis in cancer cells and thus promote their survival under such abnormal proteostasis. However, cells with such a persistent ER stress are highly susceptible to additional ER stress, and prone to cell death induction^44^. These observations imply that cells have a certain threshold of ER stress tolerance, and excessive ER stress leads to cell death. Recent studies suggested that ER stress is a key player in TNBC survival and proliferation, and may be the long searched hallmark of TNBC^45^. Accumulating evidence demonstrates that basal ER stress and UPR are typically activated in TNBC, and such non-lethal ER stress and UPR response promote oncogenic signaling pathways in TNBC cells by activating XBP1 and other UPR downstream transcription factors^46^. Our meta-analysis data showing the high levels of ER molecular chaperones PDIA4 and PDIA6 in TNBC tumors also support this notion that TNBC tumors have adapted to the sustained ER stress condition. Based on the dual-sided role of ER stress in cancer cell survival, it is possible to design ER-stress-mediated therapeutic strategies by either promoting ER stress to induce TNBC cell death or inhibiting ER stress and UPR signaling in TNBC cells to block TNBC progression. The results presented in this report demonstrate that the ER translocon inhibitor ipomoeassin F is an exciting candidate for the former approach.

In conclusion, the present study unveiled a new pharmacological activity of the natural product ipomoeassin F. We demonstrate that ipomoeassin F selectively inhibits TNBC cell growth via blocking the translocation of the ER molecular chaperones PDIA6 and PDIA4 to the ER, leading to ER stress, UPR activation and autophagy induction. Our results provide an idea that PDIA6/PDIA4 and Sec61α are attractive drug targets for TNBC targeted therapy, and that ipomoeassin F is a novel lead with excellent potency and specificity to these targets in TNBC cells.

## Supplementary Material

Supplementary figures and tables.
