# Supplementary material for: ER translocon inhibitor ipomoeassin F inhibits triple-negative breast cancer growth via blocking ER molecular chaperones
Source: Int J Biol Sci. 2023 Jul 31;19(13):4020–35. doi: 10.7150/ijbs.82012 (PMC10496495; doi:10.7150/ijbs.82012)
Supplement: Supplementary file 1 — Supplementary figures and tables. [file ijbsv19p4020s1.pdf]

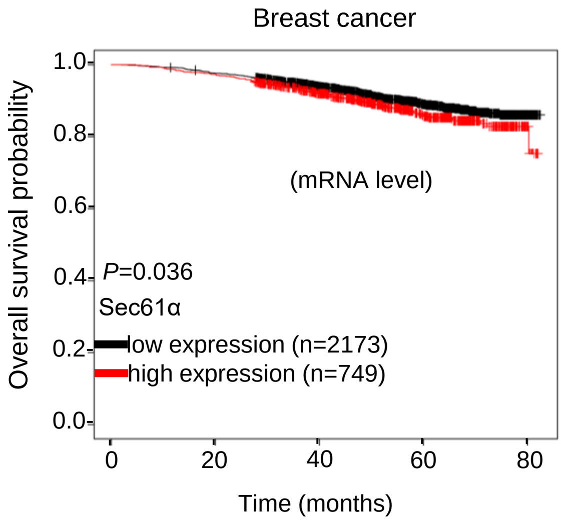

Supplementary Figure S1. The clinical association between sec61α mRNA expression level and all types of breast cancer patients' overall survival probability using Kaplan-Meier plot. The log-rank test was used for statistical analysis of differences between the high and low sec61α mRNA expression groups. $P < 0.05$ represents a significant difference between two analyzed groups.

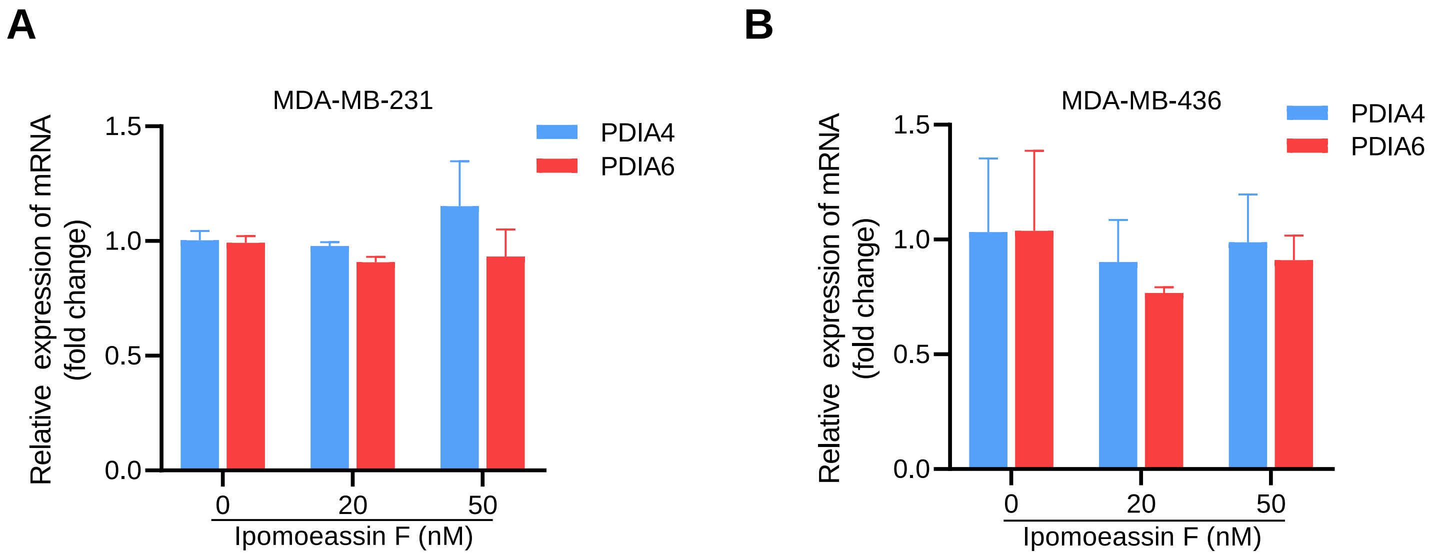

Supplementary Figure S2. Effect of ipomoeassin F on mRNA levels of PDIA6 and PDIA4 in TNBC cells. Relative mRNA levels of PDIA4 and PDIA6 after ipomoeassin F (0, 20, 50 nM) treatment for 48 h in MDA-MB-231 **(A)** and MDA-MB-436 **(B)** cells.

Supplementary Table S1

LC_{50} and TGI_{50} value of Ipomoeassin F.

Cell types	Cell lines	LC_{50}	TGI_{50}	LC_{50}/TGI_{50}
Lung cancer	A549/ATCC	0.0001	6.45654E-05	1.548816619
	EKVX	0.0001	0.0001	1
	HOP-62	8.31764E-05	4.57088E-06	18.19700859
	HOP-92	6.91831E-05	1.99526E-06	34.67368505
	NCI-H226	0.0001	1.07152E-05	9.332543008
	NCI-H23	0.0001	9.54993E-05	1.047128548
	NCI-H322M	0.0001	0.0001	1
	NCI-H460	0.0001	3.71535E-05	2.691534804
	NCI-H522	2.45471E-05	2.13796E-06	11.48153621
Colorectal cancer	COLO 205	0.0001	1.44544E-06	69.18309709
	HCC-2998	0.0001	0.0001	1
	HCT-116	6.76083E-05	1.12202E-05	6.025595861
	HCT-15	0.0001	0.0001	1
	HT29	2.39883E-06	1.65959E-07	14.45439771
	KM12	0.0001	0.0001	1
	SW-620	0.0001	3.63078E-05	2.754228703
CNS cancer	SF-268	10000	1.94984E-05	512861384
	SF-295	7.76247E-05	1.09648E-06	70.79457844
	SF-539	1.99526E-05	1.69824E-06	11.74897555
	SNB-19	0.0001	1.54882E-05	6.45654229
	SNB-75	0.0001	7.4131E-07	134.8962883
	U251	4.36516E-05	3.98107E-06	10.96478196
Ovarian cancer	IGROV1	0.0001	1.86209E-05	5.370317964
	OVCAR-3	0.0001	1.1749E-05	8.511380382
	OVCAR-4	0.0001	7.24436E-05	1.380384265
	OVCAR-5	0.0001	4.0738E-05	2.454708916
	OVCAR-8	0.0001	0.0001	1
	NCI/ADR-RES	0.0001	2.95121E-07	338.8441561
	SK-OV-3	0.0001	1.07152E-05	9.332543008
Renal cancer	786-0	8.12831E-05	1.04713E-05	7.762471166
	A498	0.0001	9.33254E-06	10.71519305
	RXF 393	5.24807E-05	3.31131E-06	15.84893192
	SN12C	0.0001	1.99526E-05	5.011872336
	TK-10	0.0001	7.76247E-06	12.88249552
	UO-31	0.0001	1.94984E-05	5.12861384
Prostate cancer	PC-3	0.0001	1.94984E-05	5.12861384
	DU-145	0.0001	0.0001	1
Breast cancer	MCF7	0.0001	1.65959E-05	6.025595861
	HS 578T	0.0001	2.75423E-05	3.630780548
	BT-549	0.0001	1.77828E-05	5.623413252
	T-47D	7.76247E-05	5.62341E-07	138.0384265
	MDA-MB-468	3.63078E-05	7.58578E-07	47.86300923
	MDA-MB-231	3.01995E-06	0.00000001	301.995172

Supplementary Table S2
Information of antibodies used in this study.

Antibodies	Suppliers	Cat. No	Dilution for WB	Dilution for IF
PDIA6	Proteintech	18233-1-AP	1:1000	1:100
ERp72(PDIA4)	Cell Signaling Technology	#2798s	1:1000	/
Calnexin	Proteintech	10427-2-AP	1:2000	/
GRP78/Bip	Proteintech	11587-1-AP	1:1000	/
eIF2α	Cell Signaling Technology	#5324s	1:1000	/
Pho-eIF2α	Cell Signaling Technology	#3597s	1:1000	/
Cleaved Caspase3	Cell Signaling Technology	#9664s	1:1000	/
LC3B	Cell Signaling Technology	#2775s	1:1000	/
m-TOR	Cell Signaling Technology	#2983	1:1000	/
P-m-TOR	Cell Signaling Technology	#5536	1:1000	/
GAPDH	Santa Cruz Biotechnology	sc-365062	1:2000	/
α-Tubulin	Santa Cruz Biotechnology	sc-5286	1:2000	/
Goat anti-mouse, HRP linked	Santa Cruz Biotechnology	sc-2005	1:2000	/
Goat anti-mouse, HRP linked	Santa Cruz Biotechnology	sc-2004	1:2000	/
Alexa Fluor 488 donkey anti-mouse IgG (H+L)	Thermo Scientific	A21202	/	1:1000
Alexa Fluor 594 donkey anti-rabbit IgG (H+L)	Thermo Scientific	A21207	/	1:1000

Supplementary Table S3

The sequence information of the siRNAs used in this study.

Target	siRNA sequence
PDIA4	Sense-5'-GGUGUUUCACUUCAUGCUCUGAATA-3’<br>Antisense-5'-UAUUCAGAGCAUGAAGUGAAACACCAA-3'
PDIA6	Sense-5'-GGAUGCUACAGUCAAUCAGGUUCTG-3’<br>Antisense-5'-CAGAACCUGAUUGACUGUAGCAUCCAC-3'

Supplementary Table S4

The sequence information of the RT-qPCR primers used in this study.

**Targets**	**Primer sequence**
GAPDH-Forward	5'-GTGGACCTGACCTGCCGTCT-3'
GAPDH-Reverse	5'-GGAGGAGTGGGTGTCGCTGT-3'
PDIA4-Forward	5'-GGCAGGCTGTAGACTACGAG-3'
PDIA4-Reverse	5'-TTGGTCAACACAAGCGTGACT-3'
PDIA6-Forward	5'-GGACACTGCAAAAACCTAGAGC-3'
PDIA6-Reverse	5'-CCAGAACCTGATTGACTGTAGCA-3'
XBP1(h)-Forward	5'-TTGCTGAAGAGGAGGCGGAAG-3’
XBP1(h)-Reverse	5'-GGTCCAAGTTGTCCAGAATGC-3'
